# Emergence of Pandemic Clonal Lineage Sequence Types 131 and 69 of Extraintestinal Escherichia coli as a Cause of Meningitis: Is It Time To Revise Molecular Assays?

**DOI:** 10.1128/spectrum.03274-22

**Published:** 2023-02-14

**Authors:** Maria Antonia De Francesco, Anna Bertelli, Silvia Corbellini, Erika Scaltriti, Francesco Risso, Roberto Allegri, Giorgio Tiecco, Francesco Castelli, Arnaldo Caruso

**Affiliations:** a Institute of Microbiology, Department of Molecular and Translational Medicine, University of Brescia, ASST Spedali Civili, Brescia, Italy; b Risk Analysis and Genomic Epidemiology Unit, Istituto Zooprofilattico Sperimentale della Lombardia e dell’Emilia Romagna, Parma, Italy; c Neonatalogy and Neonatal Intensive Care Unit, ASST Spedali Civili, Brescia, Italy; d Division of Infectious and Tropical Diseases, ASST Spedali Civili, Brescia, Italy; e Department of Clinical and Experimental Sciences, University of Brescia, Brescia, Italy; Institut National de Santé Publique du Québec

**Keywords:** K1 capsule, lineage, meningitis, uropathogenic

## Abstract

Two Escherichia coli strains, respectively responsible for neonatal and adult meningitis, were isolated and their phenotypic antibiotic susceptibility and genomic features characterized by whole-genome sequencing (WGS). Multiplex real-time PCR targeting the principal microorganisms involved in meningitis etiology failed to identify either isolate. Afterwards, matrix-assisted laser desorption ionization–time of flight (MALDI-TOF) mass spectrometry was used to identify the isolates as E. coli strains. Genomic analysis showed that they belonged to sequence types 131 and 69 (ST131 and ST69). Neither of the isolates harbored the K1 capsular antigen or belonged to other capsular serotypes, but they shared different virulence factors, including *ibe* genes, responsible for invasion of brain endothelial cells.

**IMPORTANCE** The extraintestinal pathogenic Escherichia coli group is characterized by the presence of uropathogenic E. coli (UPEC), sepsis-associated E. coli (SEPEC), and neonatal meningitis E. coli (NMEC). All of these members exhibit many virulence factors, such as lipopolysaccharides, toxins, iron acquisition factors, invasins, fimbriae, and capsules. Urinary infections are the most common infections caused by this group, followed by globally increasing numbers of both community- and nosocomially acquired bloodstream infections, associated with considerable patient morbidity and mortality. Some lineages tend to become dominant; in addition to enhanced fitness, this epidemiological success stems from increased virulence, antibiotic resistance, gut colonization, and greater host-to-host transmission. Our results underline the importance of continuous surveillance of these new emerging lineages and the need to develop new meningitis molecular assay panels able to identify them.

## OBSERVATION

Escherichia coli meningitis is frequently described in newborns with high mortality, especially in premature and low-weight infants (1). The strains mostly associated with neonatal meningitis belong to sequence type complex 95 (STc95) and to phylogroup B2. They share an important virulence factor, i.e., the presence of the K1 capsular antigen. Among adults, community-acquired meningitis due to Gram-negative rods is a rare event, where E. coli represents 0.5% to 3% of the meningitis-causing agents, and data about their genomic characteristics are few (2).

Here, for the first time in Europe, to the best of our knowledge, we report a neonatal meningitis case with a fatal outcome caused by an ST131 E. coli strain. Then, we describe the isolation of a multidrug-resistant ST69 E. coli strain causing adult meningitis. Neither strain was recognized by the molecular assay implemented (the FilmArray meningitis/encephalitis [ME] panel), which is able to detect only E. coli K1 strains. The strains were successfully identified by matrix-assisted laser desorption ionization–time of flight (MALDI-TOF) mass spectrometry and characterized by whole-genome sequencing (WGS) analysis.

The isolates were recovered from two patients admitted to the Spedali Civili Hospital, Brescia, Italy, between September 2021 and January 2022.

The first E. coli strain was isolated from a female patient born vaginally at week 40 + 1 with a birthweight of 3.06 kg and an Apgar score of 9 to 10. The newborn patient was affected by mild respiratory distress at 12 h of age and was admitted to the Neonatal Intensive Care Unit (NICU). At admission, the newborn presented with a high fever (39°C), arterial pressure of 83/41/56 mmHg, resting heart rate (RR) of 100 beats/min, and oxygen saturation (SaO_2_) level of 100%. Blood tests showed an elevated C-reactive protein (CRP) level of 71 mg/L (normal value, <5.0) and a normal level of white blood cells and platelets. Blood cultures were performed, and Gram-negative rods grew rapidly in the blood (5.9 h).

Ampicillin (300 mg every 12 h), gentamicin (12 mg per day), cefotaxime (150 mg every 12 h), and meropenem (120 mg every 12 h) were given as the recommended first-line therapy for early neonatal sepsis. Soon after admission to the NICU, the patient developed neurological signs, such as hypotonia and apnea, confirmed by cerebral echography. A lumbar puncture was performed, and analysis of the cerebrospinal fluid (CSF) showed a clear color; pleocytosis with 185 cells/μL (normal range, 50 to 100 cells/μL), comprised prevalently of polymorphonuclear cells; an increased level of proteins of 820 mg/L (normal range, 150 to 450 mg/L); and normal glucose of 53 mg/dL (normal range, 50 to 100 mg/dL). Antimicrobial therapy was continued in consideration of the antibiotic profile of the E. coli strain isolated in the CSF. Unfortunately, the clinical conditions of the newborn patient progressively deteriorated, and she died 8 days later.

The second isolate was recovered from an immunocompromised 65-year-old white woman. Her medical history reported a marginal lymphoma in remission, systemic lupus erythematosus, and autoimmune hemolytic anemia on steroid treatment. On admission, laboratory tests revealed a white blood cell count of 10.25 × 10^3^ cells/μL (reference value, 4.00 to 10.80 cells/μL), absolute neutrophilia (89%), and elevated C-reactive protein (298.5 mg/L) and procalcitonin (31 mg/dL) levels. Blood and urine culture tests were promptly performed, and considering the health care-associated infection risk factors, empirical antimicrobial treatment was started, with 4.5 g intravenous (i.v.) piperacillin-tazobactam every 6 h under suspicion of urosepsis. On day 3, an extended-spectrum β-lactamase (ESBL)-positive Escherichia coli strain susceptible to the current ongoing treatment was isolated from the blood culture. On day 7, acute clinical worsening was observed, with a high fever episode (up to 39.7°C) associated with an altered mental status (Glasgow Coma Scale [GCS] score, 13). Moreover, a single generalized seizure episode occurred, and diffuse cerebral abnormalities were recorded by the electroencephalogram. Septic meningitis was diagnosed, as the cerebrospinal fluid (CSF) showed hypoglycorrhachia, with increased protein and neutrophil levels. The antimicrobial therapy was improved with meropenem (2 g every 3 h) and vancomycin (2 g as a loading dose, then 500 mg every 6 h). The piperacillin-tazobactam was discontinued. Cerebral magnetic resonance imaging was performed, showing leptomeningitis with caudal meningoradiculitis and ventriculitis. In particular, purulent material was present in the first and second cerebral ventricles. An ESBL-positive Escherichia coli strain was isolated from the CSF culture. On day 30, antimicrobial therapy was discontinued, and soon after, the patient was discharged and transferred to a rehabilitation facility to continue the physiotherapy program already started during hospitalization.

Identification of the isolates was performed using matrix-assisted laser desorption ionization–time of flight (MALDI-TOF) mass spectrometry (bioMérieux, Florence, Italy).

Antimicrobial susceptibility testing was performed using an automated broth microdilution system (Vitek 2; bioMérieux). The Etest method (bioMérieux) was used for imipenem, meropenem, ceftazidime-avibactam, and ceftolozane-tazobactam, whereas the broth microdilution method was used for colistin, as recommended recently by the European Committee on Antimicrobial Susceptibility Testing (EUCAST) and the Clinical and Laboratory Standards Institute (CLSI). The results were interpreted according to the 2021 EUCAST guidelines (https://eucast.org).

Molecular analysis on the CSF samples was performed using the FilmArray meningitis/encephalitis (ME) panel (bioMérieux), which has the capacity to identify all the most important bacteria involved in meningitis etiology. However, it failed to identify either isolate.

The two Escherichia coli isolates (named isolate 1 and isolate 2) underwent WGS using the Illumina MiSeq platform.

PlasmidFinder 2.0 (https://cge.food.dtu.dk/services/PlasmidFinder/), SerotypeFinder 2.0 (https://cge.food.dtu.dk/services/SerotypeFinder/), and the Virulence Factor Database (VFDB) through the VFanalyzer tool (http://www.mgc.ac.cn/cgi-bin/VFs/v5/main.cgi) were used to assess the presence of plasmids, serotypes, and virulence factors, respectively.

Isolate 1 was susceptible to all the tested antibiotics, while isolate 2 showed a multidrug-resistant phenotype. It was resistant to most beta-lactam antibiotics, including broad-spectrum cephalosporins, trimethoprim/sulfamethoxazole, and ciprofloxacin, remaining susceptible to amikacin, amoxicillin-clavulanic acid, colistin, tigecycline, and carbapenems. The isolate 2 genes encoding antimicrobial resistance to different classes of antibiotics are shown in Table 1.

**TABLE 1:** Genomic characteristics of E. coli isolates

Characteristic	Data for:	
	Isolate 1	Isolate 2
Phylogenetic group	B2	D
Sequence type	131	69
K1 capsule	None	None
Serotype	O25:H4	O15:H18
*fimH* ^*a*^	682	27
Virulence score^*b*^	6	4
Antimicrobial resistance gene(s)	None	*sul2* (sulfonamide resistance); *aph(6)-Id*, *aph(3′)-Ib* (aminoglycoside resistance); *qnrS1* (quinolone resistance); *tet*(A) (tetracycline resistance); *bla*_TEM-1B_ (beta-lactam resistance)

a *fimH* refers to the allele identified for the *fimH* gene that is used for E. coli subtyping.

b A virulence score of 6 was assigned for the presence of the following genes: *fuyA*, *foc*/*sfa*, *ibeA*, *hly*, *cnf*, and *iro*, while a virulence score of 4 was assigned for the presence of the following genes: *fuyA*, *hly*, *iut*, and *iro*.

In isolate 2, two different plasmids, IncFIC(FII) (GenBank accession number AP001918) and IncX1 (EU370913), carrying the *bla*_TEM_ gene were identified, with an identity of >96%.

Whole-genome sequencing showed that the strains belonged to ST131 (phylogenetic group B2 and serotype O25:H4) and ST69 (phylogenetic group D and serotype O15:H18), respectively (Table 1).

Both E. coli isolates carried different virulence factors, as shown in Table 2. A typical serotype O18:K1 ST95 neonatal meningitis E. coli (NMEC) isolate (strain IHE3034) found in a public database (GenBank accession number NC_017628) was used as a comparison.

**TABLE 2:** Virulence factors of E. coli isolates 1 and 2 compared to a classical neonatal meningitis E. coli strain

Function	Virulence factor^*a*^	Related gene(s)	Isolate 1	Isolate 2	O18:K1 NMECST95 (IHE3034)^*b*^
Adherence	E. coli common pilus	*ecpR*	−	+	+
		*ecpA*	+	+	+
		*ecpB*	+	+	+
		*ecpC*	+	+	+
		*ecpD*	+	+	+
		*cpE*	+	+	+
	EaeH	*eaeH*	+	+	+
	F1C fimbriae	*focI*	+	−	−
		*focC*	+	−	−
		*focD*	+	−	−
		*focF*	−	−	−
		*focG*	+	−	−
		*focH*	−	−	−
	Hemorrhagic E. coli pilus	*hcpA*	+	+	+
		*hcpB*	+	+	+
		*hcpC*	+	+	+
	P fimbriae	*papX*	−	−	−
		*papG*	+	−	−
		*papF*	−	−	−
		*papE*	−	−	−
		*papK*	−	−	−
		*papJ*	−	−	−
		*papD*	+	−	−
		*papC*	+	−	−
		*papH*	+	−	−
		*papA*	−	−	−
		*papB*	−	−	−
		*papI*	+	−	−
	S fimbriae	*sfaC*	+	−	+
		*sfaB*	−	−	+
		*sfaA*	−	−	+
		*sfaD*	−	−	+
		*sfaE*	−	−	+
		*sfaF*	−	−	+
		*sfaG*	−	−	+
		*sfaS*	−	−	+
		*sfaH*	−	−	+
	Type I fimbriae	*fimB*	−	−	+
		*fimE*	+	+	+
		*fimA*	+	+	+
		*fimI*	+	+	+
		*fimC*	+	+	+
		*fimD*	+	+	+
		*fimf*	+	+	+
		*fimG*	+	+	+
		*fimH*	+	+	+
Autotransporter	Contact-dependent inhibition system	*cdiA*	+	−	−
		*cdiB*	+	−	−
	EhaB	*ehaB*	+	+	+
	EspC	*espC*	+	−	−
	Temperature-sensitive hemagglutinin	*tsH*	+	−	−
	UpaG adhesin	*upaG*/*ehaG*	+	−	+
	Cah	*cah*	−	+	+
	Immunoglobulin repeat protein	*air*/*eaeX*	−	+	−
	Vacuolating autotransporter gene	*vat*	−	+	+
Invasion	Invasion of brain endothelial cells	*ibeA*	+	−	+
		*ibeB*	+	+	+
		*ibeC*	+	+	+
	Tia/Hek	*tia*	+	−	−
Iron uptake	Heme uptake	*chuS*	+	+	+
		*chuA*	+	+	+
		*chuT*	+	+	+
		*chuW*	+	+	+
		*chuX*	+	+	+
		*chuY*	+	+	+
		*chuU*	+	+	+
	Aerobactin siderophore	*iutA*	−	+	−
		*iucD*	−	+	−
		*iucC*	−	+	−
		*iucB*	−	+	−
		*iucA*	−	+	−
	Iron-regulated element	*ireA*	+	−	−
	Iron/manganese transport	*sitA*	−	−	+
		*sitB*	−	−	+
		*sitC*	−	−	+
		*sitD*	−	+	+
	Iron/manganese transport	*iroN*	+	+	+
		*iroE*	+	+	+
		*iroD*	+	+	+
		*iroC*	+	+	+
		*iroB*	+	+	+
	Yersiniabactin siderophore	*ybtS*	+	+	+
		*ybtX*	+	+	+
		*ybtQ*	+	+	+
		*ybtP*	+	+	+
		*ybtA*	+	+	+
		*irp2*	+	+	+
		*irp+*	+	+	+
		*ybtU*	+	+	+
		*ybtE*	+	+	+
		*fyuA*	+	+	+
Toxin	Alpha hemolysin	*hlyC*	−	−	−
		*hlyA*	+	−	−
		*hlyB*	+	−	−
		*hlyD*	+	−	−
	Colicin-like Usp	*usp*	+	−	−
	Cytotoxic necrotizing factor 1	*cnf1*	+	−	−
	Hemolysin/cytolysin A	*hlyE*/*clyA*	Partial	+	−
	Cytolethal distending toxin	*cdtA*	+	−	+
		*cdtB*	+	−	+
		*cdtC*	−	−	+
Non-LEE-encoded T3SS^*c*^	Only genes present in our sequences	*espL1*	−	+	−
		*espL4*	−	+	−
		*espR1*	−	+	−
		*espX1*	−	+	−
		*espX4*	−	+	−
		*espX5*	−	+	−
		*espY1*	−	+	−
		*espY2*	−	+	−
		*espY3*	−	+	−
Secretion system	ACE T6SS (only genes present in our sequences)	*aec32*	−	+	+
		*aec31*	−	+	+
		*aec30*	−	+	+
		*aec29*	−	+	+
		*aec28*	−	+	+
		*aec27*/*clpV*	−	+	+
		*aec26*	−	+	+
		*aec25*	−	+	+
		*aec24*	−	+	+
		*aec22*	−	+	+
		*aec19*	−	+	+
		*aec18*	−	+	+
		*aec17*	−	+	+
		*aec16*	−	+	+
		*aec15*	−	+	+
		*aec14*	−	−	+
		*aec11*	−	−	+
		*aec8*	−	−	+
		*aec7*	−	−	+

a E. coli common pilus (ECP), hemorrhagic E. coli pilus (HCP), contact-dependent inhibition (CDI) system, invasion of brain endothelial cells (Ibes).

b NMEC, neonatal meningitis E. coli. Light gray shading indicates the absence of a gene; dark gray shading indicates the gene’s presence.

c Non-LEE-encoded T3SS (type 3 secretion system): only genes present in our sequences.

We observed that E. coli isolate 1, which belongs to the B2 group, carried a deletion in the *clyA* gene, while E. coli isolate 2, which belongs to the D group, harbored the complete *clyA* gene sequence. In particular, coverage of the *clyA* gene for E. coli isolate 1 was 45.4%, while coverage of the *clyA* gene for E. coli isolate 2 was 100%.

These findings confirm the results of Enow et al. (3) showing that deleted forms of the gene *clyA* are found exclusively in E. coli phylogroup B2 strains, including enteropathogenic E. coli and extraintestinal pathogenic E. coli, while the intact gene is relatively conserved in strains from other phylogroups.

**FIG 1:**
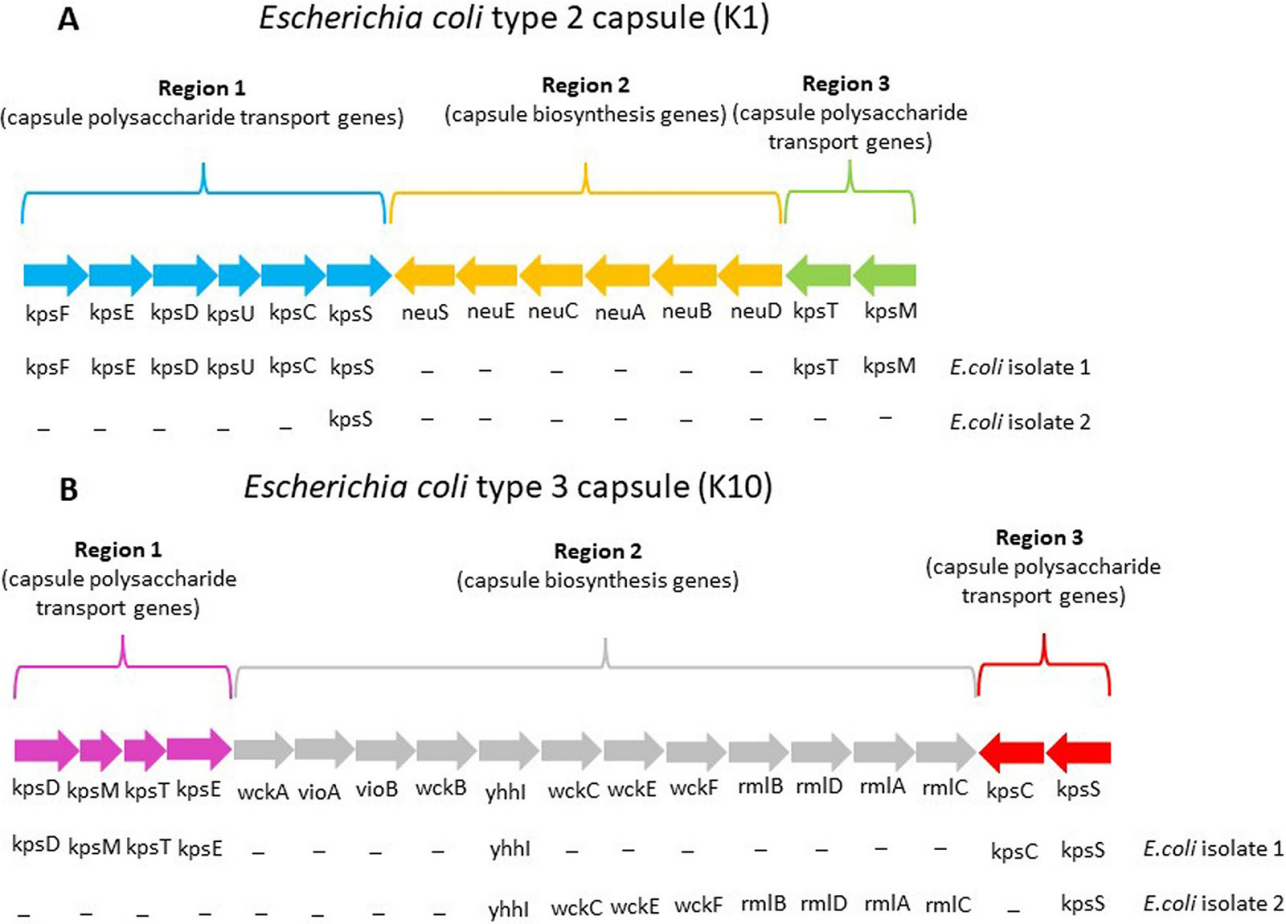
Capsule genetic locus of capsular groups 2 and 3 of E. coli. The presence of each capsular gene was checked manually by mapping the raw sequencing reads of each E. coli isolate to the capsular type reference sequence using Bowtie2, implemented in Geneious 11.1.5 software (Biomatters Ltd., Auckland, New Zealand). Capsule reference sequences: GenBank accession number MZ339217 (K1 capsule); MK278915.1 (K10 capsule).

Of note, neither of them harbored the K1 capsular genes or other capsular genes, as shown in Fig. 1. Virulence scores of 6 and 4 were assigned to isolates 1 and 2, respectively, according to the presence or absence of 11 genes (K1, *fuyA*, *papGII*, *papGIII*, *foc*/*sfa*, *hra*/*hek*, *ibeA*, *hly*, *cnf1*, *iut*/*iuc*, and *iro*) (4) (Table 1). Several recent studies showed a linkage between some extraintestinal pathogenic E. coli strains and meningitis development (4–8). Classically, NMEC strains belong to the capsular serotype K1, in particular to serotypes O18:K1 and to O7:K1, and to ST95. The presence of the capsule, together with other virulence factors, such as outer membrane proteins, siderophores, adhesins, and invasion proteins, was considered essential to permit the crossing of the blood-brain barrier.

However, our results, similarly to those reported in other recent studies (4–8), show that other virulence factors, also in the absence of the K1 capsular antigen, might be important for brain penetration of hematogenous E. coli strains circulating at high levels. In particular, it was shown that Ibe proteins might mediate the invasion of brain microvascular endothelial cells (BMECs) by linking to a transmembrane protein present in them (Caspr1). The genes coding for Ibe proteins were detected in both our isolates. The absence of the K1 capsule is a crucial point for diagnosis because the currently available molecular multiplex assays recognize only E. coli K1 strains, leading, as in our experience, to a misleading false-negative result with an important therapy delay.

Therefore, genomic characterization of these new emerging non-K1 or K-negative E. coli strains can help to update new multiplex PCR assays, including primers/probes able to detect all E. coli strains independently from the capsular genes for a more reliable meningitis diagnosis.

### Ethical approval.

The samples were taken as part of routine investigation and used anonymously. Formal consent was obtained from the parents or relatives for both cases.

### Data availability.

All E. coli isolates sequenced in this study can be found in the ENA under project number PRJEB55107.
